# ECLAT based association rule mining for advancing workplace mental health and organizational insights

**DOI:** 10.1038/s41598-026-41702-0

**Published:** 2026-03-18

**Authors:** Aman Ullah, Sana Ashraf, Eman Abdullah Aldakheel, Muhammad Abdullah, Doaa Sami Khafaga

**Affiliations:** 1 Research Institute of Business Analytics and SCM, College of Management, Shenzhen University, Shenzhen, 518060 Guangdong China; 2 College of Computer Science and Software Engineering, Shenzhen University, Shenzhen, 518060 Guangdong China; 3 Department of Computer Sciences, College of Computer and Information Sciences, Princess Nourah bint Abdulrahman University, P.O. Box 84428, Riyadh, 11671 Saudi Arabia; 4 Faisalabad Business School, National Textile University, Faisalabad, Pakistan

**Keywords:** Workplace well-being, Mental health patterns, Organizational policy, Employee productivity, ECLAT algorithm, Association rule mining, Mathematics and computing, Psychology, Psychology

## Abstract

This study explores the application of the equivalence class transformation (ECLAT) algorithm for frequent itemset mining and association rule discovery in the analysis of employee mental health data within the workplace. As mental health continues to be recognized as a crucial factor influencing workplace productivity, understanding the factors that impact employee well-being is of paramount importance. To address this, we apply the ECLAT algorithm, a technique known for its efficiency in handling large datasets through vertical transaction representation and depth-first search methods. Unlike traditional algorithms, ECLAT minimizes computational complexity and overhead, allowing for the rapid identification of frequent itemsets. By analyzing the dataset, the ECLAT algorithm identifies the most frequent combinations of these factors, and association rules are derived to uncover the relationships between various mental health elements. These rules are assessed using support, confidence, and lift metrics to determine their significance. The results offer critical insights into the influence of workplace policies on employee mental health, providing a foundation for organizations to create more supportive environments that promote mental well-being. Ultimately, this research contributes to advancing workplace well-being initiatives, fostering a mentally healthy workforce, and driving organizational success.

## Introduction

Mental health in the workplace has become a critical area of concern for organizations globally^1,2^. As awareness of the link between mental health and workplace productivity increases, understanding the factors that influence employees’ mental well-being is paramount^3–5^. However, many organizations rely on surface-level approaches, such as generic wellness programs or stress management workshops, which fail to address the root causes of mental health issues^6,7^. Traditional methods of data analysis, such as alone simple statistical surveys or qualitative interviews^8–10^, often fail to uncover the intricate patterns and relationships between various workplace factors and employee well-being. These methods lack the ability to handle large volumes of data and reveal deeper, hidden connections that could drive actionable policy change. This challenge is compounded by a lack of leadership commitment and inadequate training in mental health literacy, which leads to poorly implemented initiatives^11^. Additionally, the evolving nature of work, including remote and hybrid models, introduces new stressors like social isolation, digital fatigue, and blurred work-life boundaries. Identifying and understanding the complex interactions between these factors remain a significant challenge, underscoring the urgent need for more sophisticated, data-driven approaches to workplace mental health.

One of the primary obstacles is the lack of advanced technological tools capable of efficiently analyzing the complex, multifaceted nature of mental health data. Furthermore, many organizations lack access to advanced analytics and data mining techniques that could provide a more comprehensive understanding of employee mental health^6,7^. Without such tools, organizations are left with incomplete or oversimplified views of the factors that influence mental health outcomes. For instance, while a survey may indicate that a high percentage of employees report feeling stressed or overwhelmed, it may not offer insights into the specific combinations of factors (such as the lack of mental health resources, poor workplace culture, or long working hours) that contribute to this stress^12–14^. This signals the importance of adopting more efficient and robust techniques capable of uncovering meaningful patterns and insights.

Several popular data mining and machine learning techniques, such as the Apriori algorithm^15–19^, FP-growth^20^, decision trees^21–23^, and neural networks^24,25^, have been employed to uncover patterns and relationships in survey datasets. AdaBoost algorithm was also used for predictive analysis of mental health conditions^26^. However, each of these methods comes with its own set of limitations. For instance, the Apriori algorithm^15,18,27^, one of the most widely used for frequent itemset mining, operates by iteratively generating candidate itemsets and pruning those that do not meet the minimum support threshold. While effective for small to medium-sized datasets, its computational complexity grows exponentially with larger datasets due to the need for multiple scans and extensive candidate generation. This becomes a significant bottleneck when analyzing comprehensive employee mental health surveys, where the sheer volume of data can overwhelm traditional algorithms. Decision trees^21,23^, another common approach in machine learning, are often used to identify relationships between various features and outcomes. While they are relatively easy to interpret and can handle both categorical and continuous data, the intricate interactions between factors such as workplace culture, employee behavior, and mental health resources might lead to overly complex trees that fail to generalize well to new data. Neural networks, particularly deep learning models^24,25,28,29^, have shown significant success in handling large and complex datasets. However, these models require substantial computational resources and large amounts of labeled data to achieve accurate predictions. Moreover, the lack of interpretability of neural networks^30^ can make them challenging, where understanding the relationships between various factors is crucial for actionable insights and policy development. These technological approaches, while useful in certain contexts^31^, have inherent gaps when it comes to efficiently processing large-scale, high-dimensional datasets such as employee surveys, where both interpretability and computational efficiency are critical.

This research addresses this gap by leveraging the equivalence class transformation (ECLAT) algorithm^32^, a popular method for mining frequent itemsets and discovering association rules from large datasets^33–35^. By using vertical transaction representation and depth-first search techniques, ECLAT is able to identify frequent itemsets quickly without relying on extensive pruning or candidate generation, thus significantly reducing computational complexity. This ability makes ECLAT an ideal tool for uncovering patterns in large, complex datasets, where speed and interpretability are key^36–39^. Furthermore, the ability of ECLAT to generate association rules^40^ from the survey responses allows us to explore deeper connections between workplace mental health factors, such as the impact of wellness programs on treatment-seeking behaviors. This insight, achieved through the rapid identification of significant patterns, equips organizations with actionable data to refine their policies and foster a healthier workplace environment. The computational efficiency and clarity provided by ECLAT in generating interpretable, valuable results make it a game-changing and novel approach in mental health data analysis in organizations, especially when the data’s scale and complexity would overwhelm conventional methods.

While contemporary workplace dynamics continue to evolve, the primary objective of this study lies in demonstrating how to leverage ECLAT algorithm to extract actionable mental health insights from organizational survey data, using a historically grounded but widely validated dataset. Through efficient frequent survey responses mining and association rule discovery, the study aims to provide novel approach, actionable insights for improving mental health policies and practices within organizations, enhancing both the interpretability and computational efficiency of employee surveys. To the best of our knowledge, this represents a novel approach in the intersection of mental health and organizational development, offering innovative strategies to foster well-being and enhance workplace dynamics. Therefore, the research questions this study aims to answer are:

- What are the key factors and their combinations influencing employee mental health in the workplace?
- How can the ECLAT algorithm uncover meaningful patterns and relationships within large-scale employee mental health survey data?
- How can the association rules derived from employee mental health data contribute to the development of more effective workplace policies and practices?

The structure of this paper is organized as follows: Section 2 outlines the research methodology, covering the data collection process and our approach to data mining. Section 3 presents the results and discussions, while Section 4 provides the conclusion of the study.

## Research method

### Data

In this study, we analyze a 2014 survey dataset which measures various attitudes toward mental health and the prevalence of mental health disorders in the workplace, particularly within the tech industry^41^. Our dataset was acquired by an open source platform www.kaggle.com. Our primary objective is to leaverage the powerful capability of ECLAT algorithm^32^ in association rule mining and identify key factors influencing employee mental health^42^, and understand how these factors affect their emotional, psychological, and social well-being^13^. By uncovering these relationships, we aim to gain valuable insights that can guide organizations in fostering healthier workplace environments, ultimately enhancing both individual employee well-being and overall organizational productivity.

Mental health is a critical aspect of employee performance^35,43–45^, directly influencing how employees think, feel, and interact with others in the workplace^46^. It affects their decision-making capabilities, stress management^12,14,47^, and engagement with their tasks^48^. Poor mental health can lead to absenteeism, lower productivity, and strained workplace relationships. Research has consistently shown that mental health issues contribute significantly to lost workdays and reduced output^39,48^. Empirical evidence from the United States indicates that depression alone accounts for substantial economic losses, amounting to tens of billions of U.S. dollars annually, primarily due to impaired work performance and lost productive time^49^. At the global level, estimates reported by the World Health Organization show that common mental health conditions, including depression and anxiety, collectively result in approximately one trillion U.S. dollars in productivity losses each year worldwide, underscoring the significant economic burden of mental health challenges on organizations and societies^50^.

The dataset contains 27 attributes as shown in Table 1, which capture various dimensions of mental health in the workplace, including employee demographics, mental health-related attitudes, and organizational policies. These variables are crucial for understanding how mental health is perceived and managed within organizations and how these perceptions impact employees’ emotional and psychological well-being^51^. We provide a detailed overview of the key attributes/responses included in the dataset in Table 1. In this study, we focus on the factors directly related to workplace mental health and its impact on employee well-being and performance. The survey attributes selected in the dataset address various aspects of the employee’s personal experiences, the workplace environment, and the support systems available at work that may influence mental health^13,45,52^. The selected attributes can be categorized as follows:

- Access to mental health resources: This includes variables such as care options, seek help, and benefits, which are vital in understanding the support available by organization to employees in addressing their mental health concerns.
- Employer attitudes toward mental health: Variables like mentalhealthconsequence, physhealthconsequence, and work interfere reflect how the employer’s attitude and organizational policies impact employees’ mental health and their willingness to seek support.
- Workplace culture and support: This category includes factors like coworkers, supervisors, leave, and anonymity, which provide insights into how the social environment and the level of support within the workplace can influence mental health.
- Personal factors: These include family history and treatment^52^, which might affect employees’ perceptions and attitudes toward mental health.

Attributes such as age, geographic location, and open comments were excluded to avoid unnecessary complexity and keep the analysis focused on variables directly relevant to workplace mental health and the study’s objectives.

**Table 1:** Description of Survey Columns.

**Column Name**	**Description**
Timestamp	Records the time the survey data was collected.
Gender	Employee’s gender.
Age	Employee’s age.
Country	Employee’s country of residence.
State	Employee’s state (for U.S. residents).
Self Employed	Whether the employee is self-employed.
Family History	Whether the employee has a family history of mental illness.
Treatment	Whether the employee has sought treatment for a mental health condition.
Work Interfere	Whether mental health interferes with the employee’s work.
Care Options	Whether the employee knows mental health care options provided by their employer.
Wellness Program	Whether mental health is discussed in the employer’s wellness program.
Seek Help	Availability of resources for learning about mental health and seeking help.
Anonymity	Whether the employee’s anonymity is protected when using mental health services.
Leave	Ease of taking medical leave for mental health conditions.
Mental Health Consequence	Whether discussing mental health with an employer would have negative consequences.
Physical Health Consequence	Whether discussing physical health with an employer would have negative consequences.
Coworkers	Willingness to discuss mental health issues with coworkers.
No Employees	The number of employees in the employee’s company.
Remote Work	Whether the employee works remotely at least 50 percent of the time.
Tech Company	Whether the employee works for a tech company.
Benefits	Whether the employer provides mental health benefits.
Physical Health Interview	Whether the employee would discuss physical health issues in a job interview.
Mental vs Physical	Whether the employee feels mental health is treated as seriously as physical health.
Observed Consequence	Whether the employee has observed negative consequences for coworkers with mental health conditions.
Mental Health Interview	Would you bring up a mental health issue with a potential employer in an interview?
Supervisor	Would you be willing to discuss a mental health issue with your direct supervisor(s)?
Comments	Free-text field for additional comments.

#### Dataset provenance and temporal scope

The dataset used in this study originates from a workplace mental health survey conducted in 2014. As such, this study does not examine post-COVID workplace conditions, nor does it capture mental health dynamics associated with pandemic-driven changes such as prolonged remote work, hybrid arrangements, or post-pandemic organizational policies. Instead, the dataset is used as a historically grounded and widely cited benchmark to demonstrate the analytical effectiveness, interpretability, and scalability of the proposed ECLAT framework. The findings should therefore be interpreted strictly within this pre-COVID temporal context and are not intended to represent contemporary workplace mental health conditions. Accordingly, the primary contribution of this study lies in evaluating the capability of the ECLAT technique to uncover meaningful patterns and associations in large-scale organizational survey data, rather than in drawing time-specific substantive conclusions.

### Data preprocessing

The input to the ECLAT algorithm^32^ is a survey dataset, containing employee responses to various mental health-related questions, such as those concerning wellness programs, treatment-seeking behavior, and workplace stigma^41,53^. Missing values in categorical columns/attributes are handled by filling them with the most frequent category (mode) to ensure completeness of data^54^.

Each survey response is represented as a discrete categorical item in the form *attribute:value* (e.g., *work_interfere: Sometimes*, *treatment: Yes*). This representation follows standard association rule mining practice, making it directly compatible with the ECLAT algorithm, which operates on co-occurrence patterns of categorical items within transactions.

To support interpretation of these defined response categories, a pre-trained BERT model ^55^ is employed to obtain semantic representations of the *category labels themselves*. The motivation for this step is that many survey response options are linguistically expressed (e.g., “Never,” “Rarely,” “Sometimes,” “Often”) and convey graded levels of intensity that are meaningful in mental health assessment. BERT embeddings are used solely to inspect semantic proximity and ordinal coherence among these labels, thereby providing an additional interpretive lens without altering the original categorical meaning specified. For example, the response categories associated with the *work_interfere* attribute are represented as:

$$e_{\text {work\_interfere}} = \text {BertModel}(\text {``Never''})$$

$$e_{\text {work\_interfere}} = \text {BertModel}(\text {``Often''})$$

$$e_{\text {work\_interfere}} = \text {BertModel}(\text {``Rarely''})$$

$$e_{\text {work\_interfere}} = \text {BertModel}(\text {``Sometimes''})$$

These embeddings allow inspection of whether semantically related response labels form coherent groupings and expected ordinal progressions, which is particularly relevant in mental health surveys where response intensity conveys meaningful psychological information. Importantly, the embedding representations are used solely for semantic validation and interpretive support; they are not incorporated into frequency computation, transaction construction, or association rule generation.

After categorical encoding, each employee’s responses across all survey questions are grouped into a single transaction composed of discrete categorical items. These transactions constitute the direct input for frequent survey response mining.

For example, if an employee answers five questions, the transaction might look like:

{Q1_answer1, Q2_answer3, Q3_answer2, Q4_answer4, Q5_answer1}.

**Table 2:** Example Transactions/Records for Frequent Survey Responses Mining.

**Transaction ID**	**Items (Encoded Responses)**
T1	{work_interfere_Sometimes, treatment_Yes, benefits_Yes, remote_work_No}
T2	{work_interfere_Often, treatment_No, benefits_No, remote_work_Yes}
T3	{work_interfere_Sometimes, treatment_Yes, benefits_Yes, remote_work_Yes}
T4	{work_interfere_Sometimes, treatment_No, benefits_Yes, remote_work_No}

## Perception

From the Table 2, several frequent survey responses were identified in later stages by ECLAT like the first frequent survey response, work_interfere_Sometimes, benefits_Yes, appeared in three transactions/records (T1, T3, T4). This indicates that employees who report “work interference sometimes” often also receive benefits. An actionable insight from this pattern is to ensure that benefits provided by the organization include robust mental health support to address potential work interference.

### Transaction data generation

For each employee record $r$, the process of converting the record along with its survey responses into a transaction is as follows:

- For each categorical feature–value pair $c_i$ in the record (e.g., *work_interfere: Often*), the response is treated as a discrete categorical item.
- The record is transformed into a transaction $T_r$, which is represented as a set of categorical feature–value items corresponding to the responses selected by the employee.

The transaction $T_r$ for each record $r$ is represented mathematically as:

$$T_r = \{c_1, c_2, \dots , c_n\}$$

This transaction representation preserves the response structure and provides a direct and standard input format for frequent survey response mining.

### Vertical data representation

The data is then represented in a vertical format for efficient itemset mining as in^56,57^. In this representation, each categorical response item is mapped to the set of transactions in which it appears. The vertical format enables efficient computation of itemset support through set intersection operations. This mapping is stored in a dictionary. The vertical format ensures that frequent survey responses can be efficiently mined by leveraging transaction set intersections of the encoded categorical items. Building on this vertical data representation described, the ECLAT algorithm computes itemset support through depth-first intersections of transaction sets.

### ECLAT algorithm for mining frequent survey responses

Frequent Itemset Mining is an important step in analyzing data, especially for identifying patterns or relationships between different items^58^. In our research context of the employee survey, it helps uncover common combinations of responses that occur frequently across the dataset. For example, it could show patterns like “employees who know about mental health care options are more likely to seek help.”

The goal of this step is to calculate how often each combination of responses (or itemset) appears across all employee survey records, and store the responses that appear frequently. To decide what counts as “frequent,” a minimum threshold for support is set. Support measures the proportion of records in which a specific combination of responses appears. For example, if we set the minimum support to 5% (0.05), only response that appear in at least 5% of all records will be considered frequent.

For each combination of two responses, say $(i, j)$, the algorithm looks at how many records contain both $i$ and $j$ together. This is called the intersection of their transactions, and it gives us the support for that responses. The support is calculated using the formula:

$$\text {Support}(i, j) = \frac{|T_i \cap T_j|}{N}$$

Where:

- $T_i$ and $T_j$ are the sets of records that contain the responses $i$ and $j$, respectively.
- $|T_i \cap T_j|$ is the number of records where both responses $i$ and $j$ appear.
- $N$ is the total number of records (or transactions) in the dataset.

If the calculated support for specific set of responses is greater than or equal to the minimum support threshold, the response set is considered frequent and is stored for future analysis. The algorithm checks the following condition:

$$\text {if Support}(i, j) \ge \text {min\_support} \quad \text {then store frequent responses}$$

**Fig. 1:**
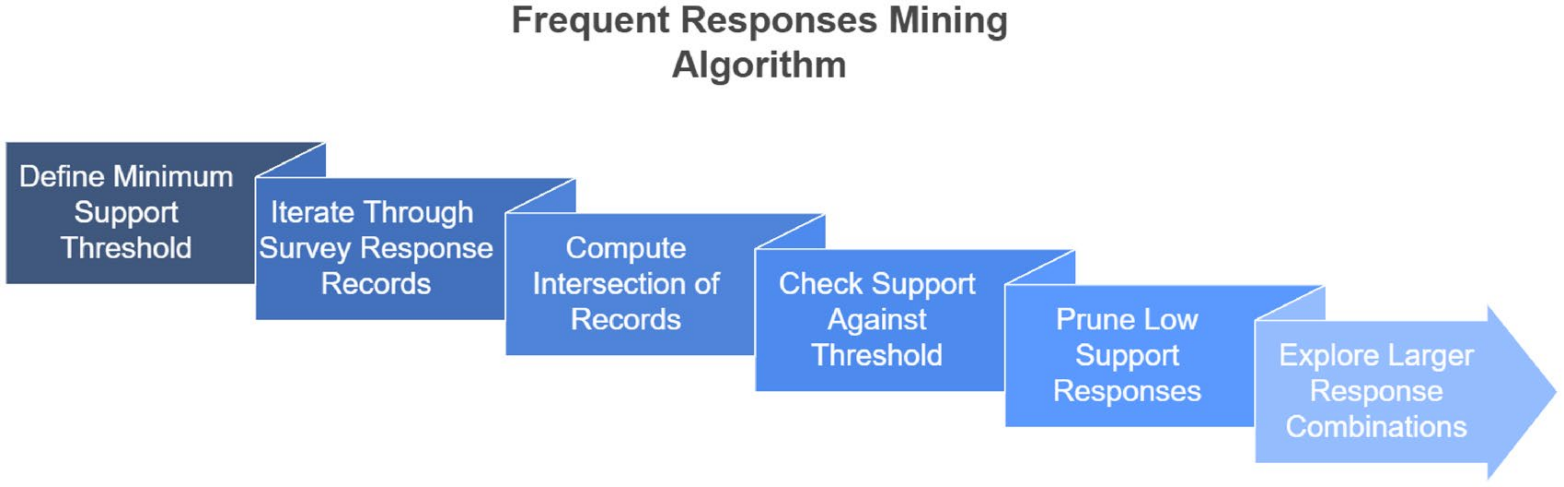
ECLAT Algorithm for Mining Frequent Survey Responses.

This process helps identify important relationships and patterns in the data that can be further explored and used to improve workplace practices or policies. The overall workflow of the ECLAT-based mining process is illustrated in Fig. 1.

### Generation of association rules

After discovering frequent response combinations, association rules are generated from them^42,59^. These rules take the form of:

$$\text {If } A \text {, then } B$$

$$\text {If } antecedent \text {, then } consequent$$

For example, a rule like: “If an employee is aware of wellness programs, then they are likely to seek help for mental health” can be derived from the itemsets found in the dataset. The antecedent represents the condition or “if” part of the rule, while the consequent represents the outcome or “then” part.

**Fig. 2:**
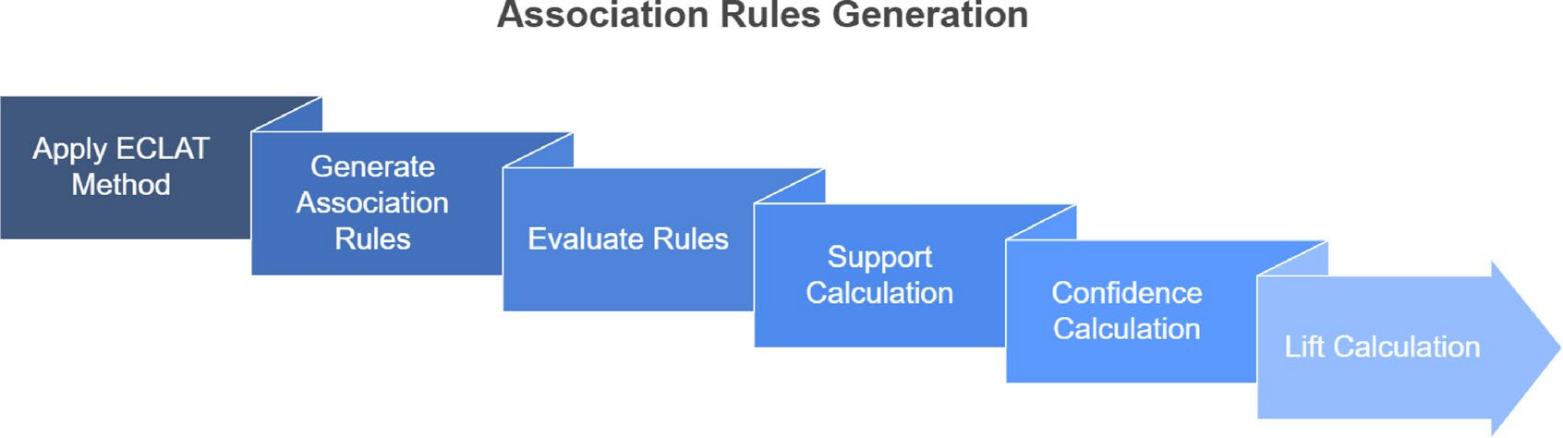
Association Rule Mining.

Our research evaluates the strength and reliability of these rules using the metrics support, confidence and lift. The process of generating association rules from frequent responses is shown in Fig. 2.

####  Support

The support of a rule is the frequency of the survey responses containing both the antecedent A and consequent B. It is defined as:

$$\text {Support}(A \cup B) = \frac{\text {count}(A \cup B)}{\text {total transactions/records}}$$

Where $A \cup B$ is the combined responses containing both antecedent and consequent. In our research, support is calculated to measure how frequently both the antecedent and consequent of a rule appear together in the dataset. For instance, a high support value for a rule like “If an employee has access to wellness programs, then they seek mental health help” means that many employees who have access to wellness programs also seek help.

#### Confidence

The confidence of a rule in our work is calculated as the probability that the consequent occurs, given that the antecedent has already occurred.

$$\text {Confidence}(A \rightarrow B) = \frac{\text {Support}(A \cup B)}{\text {Support}(A)}$$

Where $A$ is the antecedent and $B$ is the consequent. It would measure how likely it is that an employee who knows about wellness programs will seek help. The higher the confidence, the stronger the implication that knowing about wellness programs leads to seeking help.

#### Lift

Lift measures the strength of the rule by comparing its confidence to the expected confidence if $A$ and $B$ were independent:

$$\text {Lift}(A \rightarrow B) = \frac{\text {Confidence}(A \rightarrow B)}{\text {Support}(B)}$$

Lift values greater than 1 indicate that the antecedent and consequent are positively correlated (the rule has predictive power), while values less than 1 suggest a negative correlation. For instance, a high lift for the rule “If an employee has access to wellness programs, then they are likely to seek help for mental health” suggests a strong relationship between the availability of wellness programs and employees’ willingness to seek mental health support.

### Implementation details and reproducibility

**Table 3:** Implementation Details for Reproducibility.

**Aspect**	**Specification**
Original dataset size	1,259 records $\times$ 27 attributes
Preprocessing strategy	Missing categorical values imputed using mode; numerical values imputed using mean
Records removed	None (no row-wise deletion or filtering applied)
Final records analyzed	1,259 transactions
Attributes used for mining	20 categorical attributes related to workplace mental health and organizational context
Programming language	Python
Key libraries	pandas, numpy, torch, matplotlib, collections
Transaction definition	One employee record represented as a transaction of categorical responses (feature–value pairs)
Data representation	Vertical format
Frequent itemset algorithm	ECLAT (depth-first search using set intersections)
Minimum support threshold	0.05
Maximum itemset length	Not restricted (no explicit maximum length parameter applied)
Itemsets evaluated	Pairwise itemsets (implementation-level restriction for interpretability)
Association rule generation	Derived from frequent itemsets
Confidence metric	Computed for all rules (no confidence threshold applied)
Lift metric	Computed for all rules; lift values used for interpretation
Computational environment	Python-based notebook environment
Randomness / stochasticity	None (fully deterministic execution)

**Algorithm 1:**
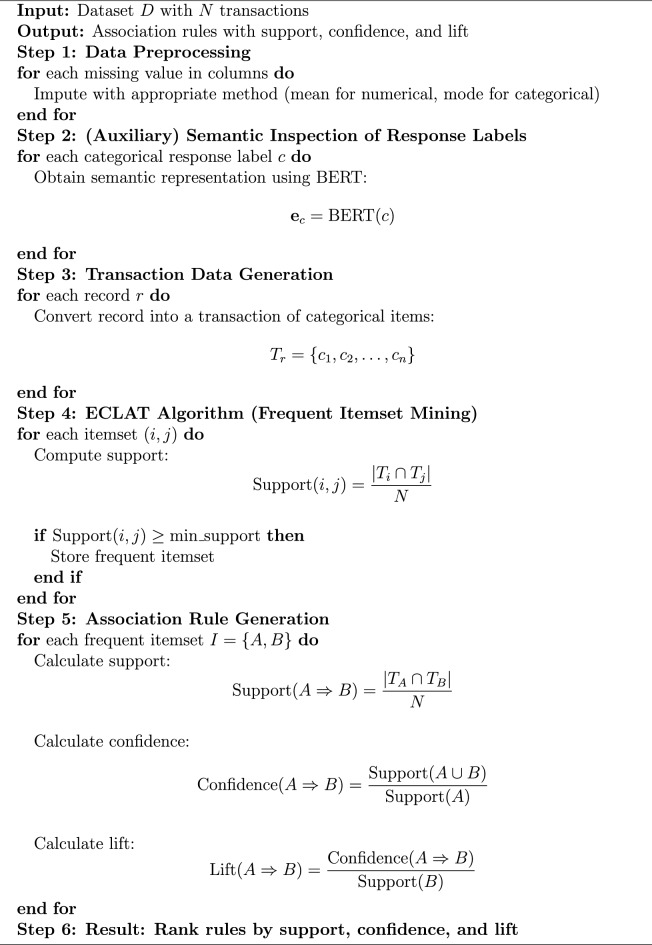
ECLAT Algorithm for Association Rule Generation

To ensure methodological transparency and reproducibility, Table 3 summarizes the key implementation details, parameter settings, and execution choices used in the ECLAT-based analysis. All reported settings correspond directly to the experimental implementation and enable exact replication of the results.

## Results and discussions

### Analysis of frequent response combinations

**Fig. 3:**
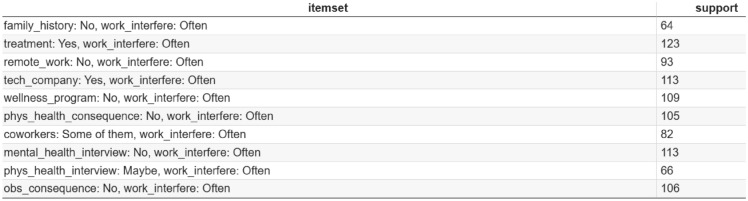
This table displays some of the random frequent combination of responses by employees to mental health-related survey questions along with their respective support values. The support value indicating the number of transactions (employee records) in which the combination appears.

Frequent response combinations extracted using the ECLAT algorithm reveal recurring patterns in how employees experience and respond to workplace mental health challenges. Figure 3 summarizes representative frequent itemsets along with their support values, indicating the proportion of employee records in which each response combination appears. These frequent response patterns shed light on employee experiences and provide a foundation for informed organizational decision-making.

Several high-support patterns involve the variable “work_interfere: Often”, highlighting frequent mental health–related disruptions to work performance. Notably, the combination “treatment: Yes, work_interfere: Often” exhibits one of the highest support values, suggesting that employees experiencing persistent work interference due to mental health issues are more likely to seek professional treatment. This pattern underscores the importance of early identification of work-related mental health strain and the availability of accessible treatment resources. Other frequent combinations, such as “wellness_program: No, work_interfere: Often” and “mental_health_interview: No, work_interfere: Often”, point to structural gaps in organizational mental health initiatives. These patterns suggest that the absence of wellness programs and mental health discussions during recruitment or onboarding may contribute to unresolved mental health challenges that interfere with work. From a managerial perspective, these findings highlight the potential value of integrating wellness programs and open mental health dialogue into organizational practices.

While these observations are valuable, they provide only an overview of commonly co-occurring responses, they do not capture directional relationships between variables. To uncover such relationships and assess conditional dependencies among workplace mental health factors, the next subsection extends the analysis using association rule mining.^40,60^. This deeper analysis enables organizations to develop more targeted and effective interventions to support employee mental health and well-being.

### Association rules discovery analysis

**Fig. 4:**
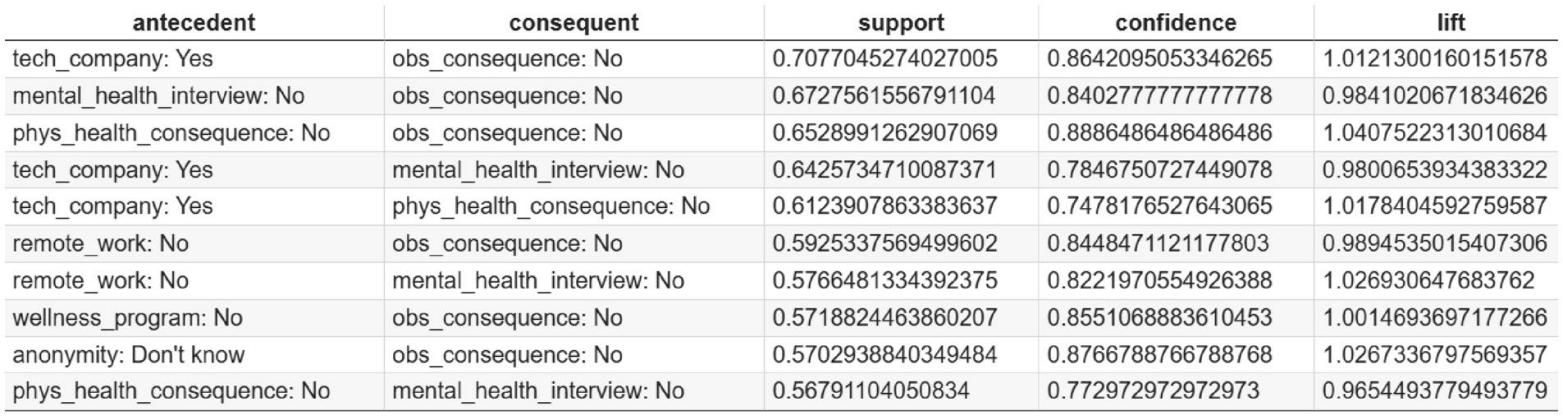
Association Rules Ranked by Support.

**Fig. 5:**
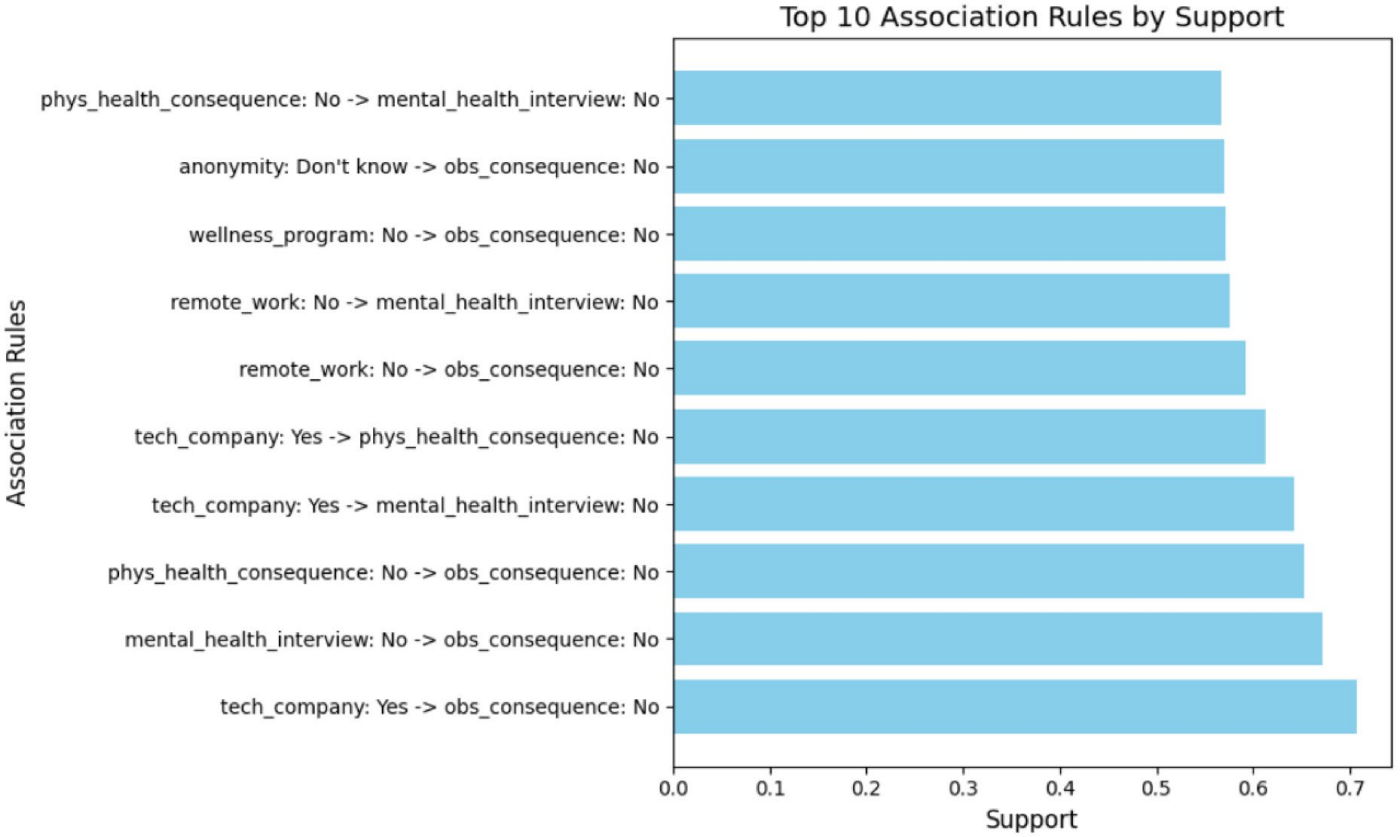
Top 10 association rules ranked by support. The x-axis represents support values computed as the proportion of employee records in which each rule occurs. Higher values indicate more prevalent co-occurring mental health response patterns.

**Fig. 6:**
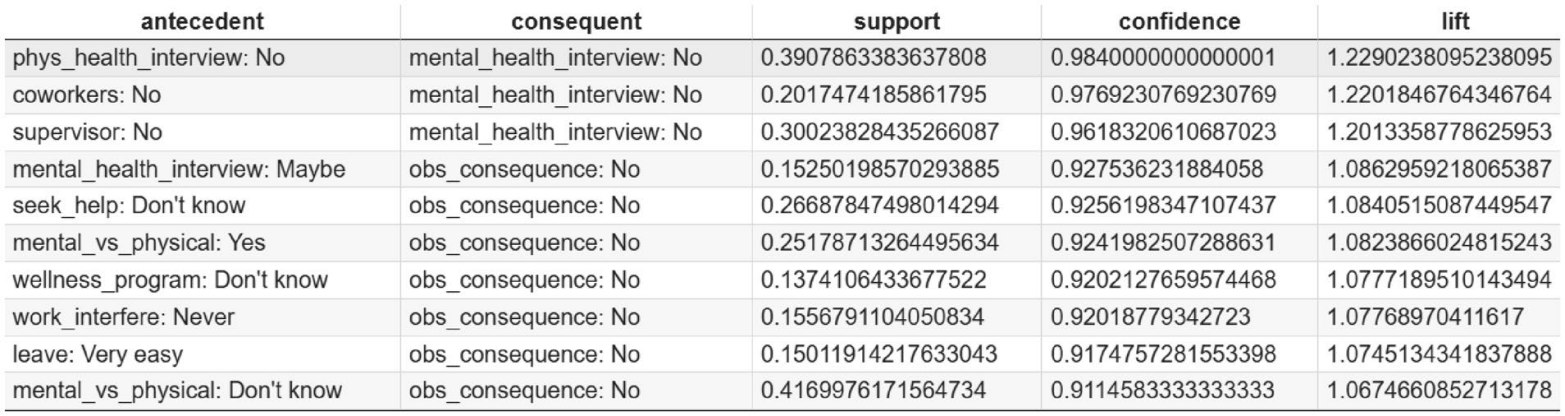
Association Rules Ranked by Confidence.

**Fig. 7:**
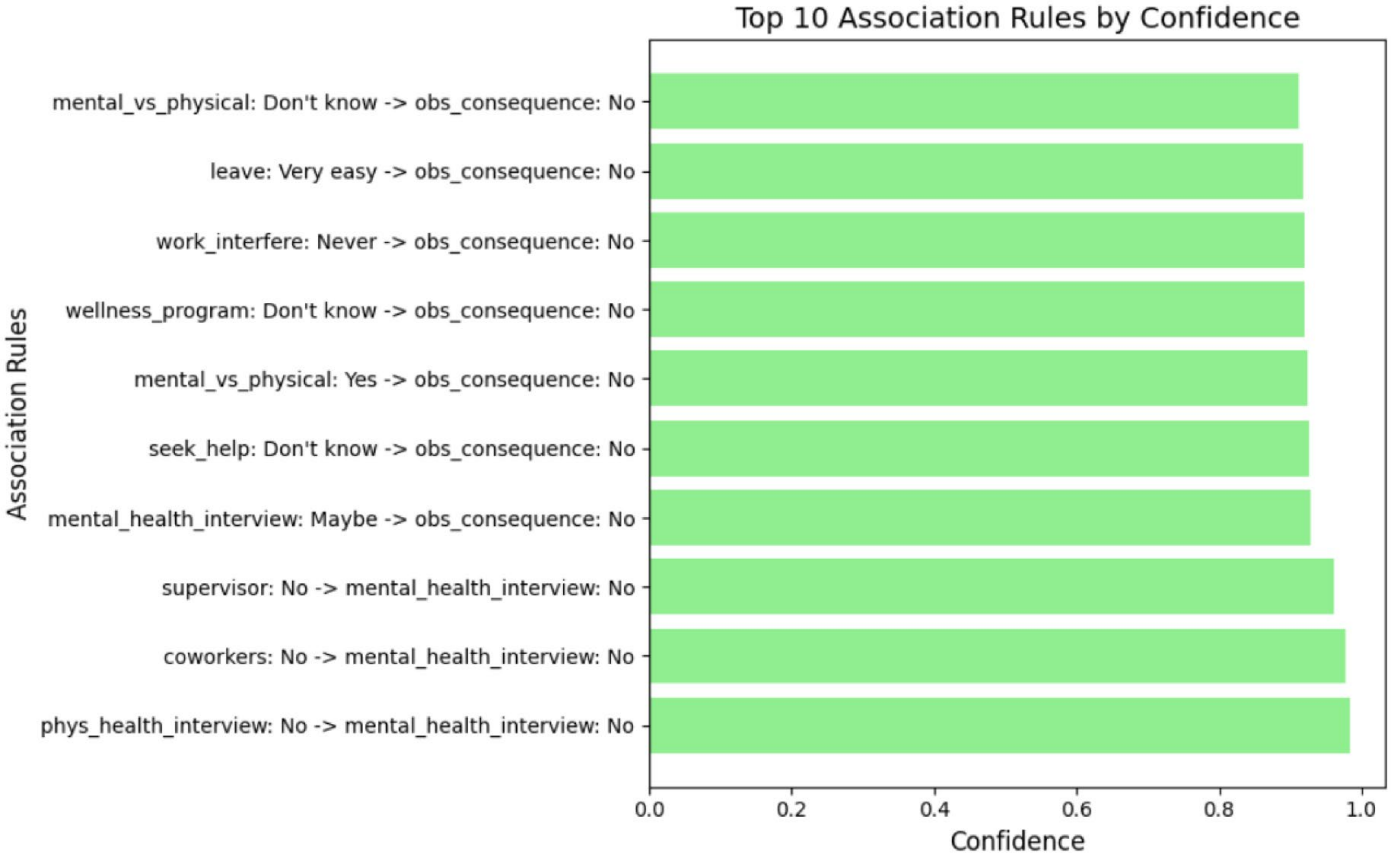
Top 10 association rules ranked by confidence. The x-axis represents confidence values, defined as the conditional probability that the consequent occurs given the antecedent. Higher values indicate stronger conditional associations between mental health response patterns.

**Fig. 8:**
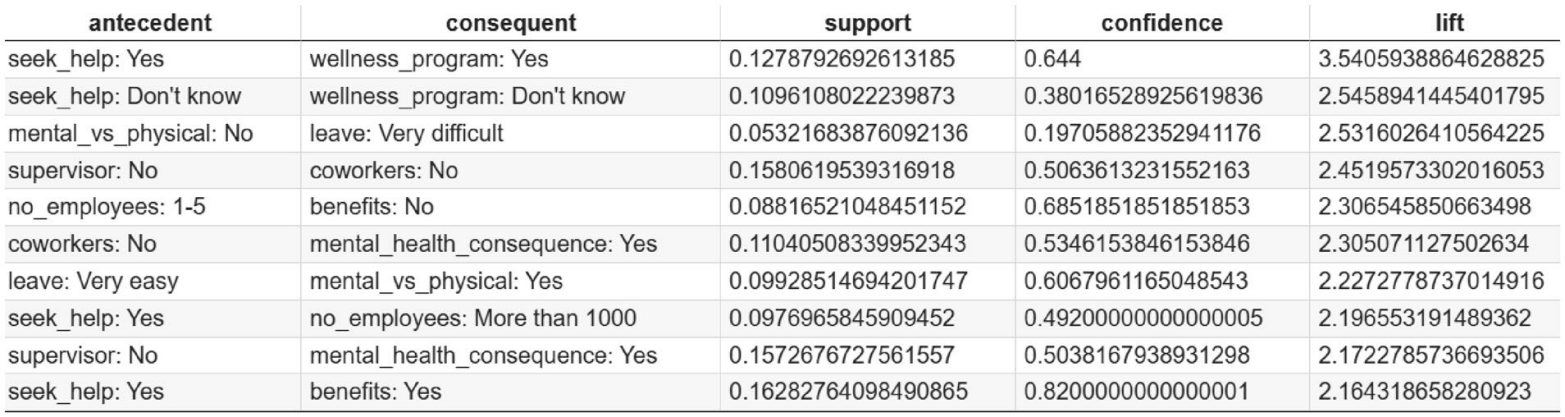
Association Rules Ranked by Lift.

**Fig. 9:**
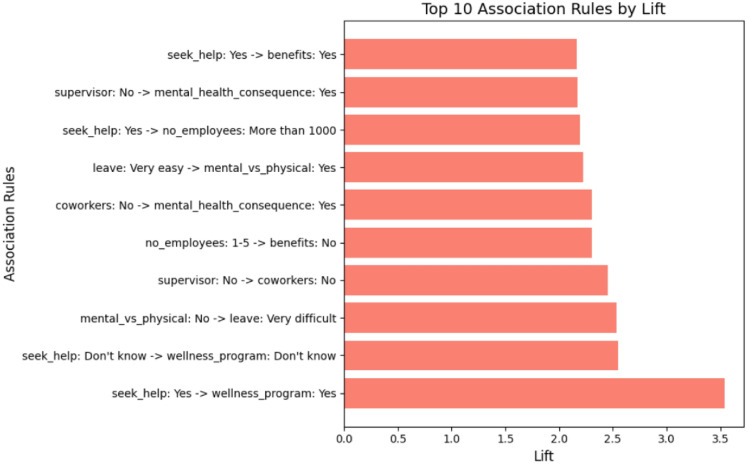
Top 10 association rules ranked by lift. The x-axis represents lift values, which quantify the strength of association between antecedent and consequent relative to their expected co-occurrence under statistical independence. Lift values greater than one indicate positive associations.

Based on the association rules presented in Figs. 4, 5, 6, 7, 8 and 9, which have been sorted according to the metrics of support, confidence, and lift, the following is a detailed analysis of some discovered results of relationships.

#### Association rule sorted by support

The association rules derived from frequent response patterns and ranked by support, as shown in Figs. 4 and 5, reveal prominent co-occurrence relationships among workplace mental health factors. The rule “tech_company: Yes $\rightarrow$ obs_consequence: No” has the highest support of 0.7077, suggesting that employees working in tech companies are more likely to report the absence of observed negative consequences for coworkers with mental health issues. The high confidence (0.8642) and lift (1.0121) values further indicate that this association is both strong and meaningful, implying that tech companies may have negative outcomes associated with mental health in comparison to other industries. This is a critical insight, as it shows that tech companies might be more progressive in fostering a supportive environment for mental health, leading to reduced perceived consequences for employees with mental health issues.

Additional rules highlight the interplay between physical health perceptions, anonymity, and mental health disclosure. For example, the rule “phys_health_consequence: No $\rightarrow$ mental_health_interview: No” (support: 0.6529, confidence: 0.8886, lift: 1.0405) indicate a broader trend where employees who are comfortable discussing physical health issues may also be more likely to feel comfortable addressing mental health. However, those who don’t perceive a negative impact of discussing physical health issues might still not be as open to addressing mental health concerns, possibly reflecting a lack of mental health awareness. Similarly, the rule “anonymity: Don’t know $\rightarrow$ mental_health_interview: No” (support: 0.5702, confidence: 0.8767, lift: 1.0267) underscores the importance of perceived confidentiality. Employees who are uncertain about the anonymity of mental health discussions are less likely to raise such issues during interviews, emphasizing the critical role of trust and transparent communication in encouraging mental health disclosure.

From an organizational perspective, these findings highlight actionable priorities. Clear communication regarding confidentiality, explicit inclusion of mental health topics in wellness initiatives, and normalization of mental health discussions—particularly during recruitment and onboarding—may reduce perceived risks associated with disclosure. These results also align with prior evidence emphasizing the role of workplace policies, including flexible work arrangements such as remote work^43^, and structured wellness programs in shaping employee mental health perceptions. While support provides valuable insights into the frequency of these associations, further evaluation of rules based on other metrics, such as confidence and lift, could provide a deeper and strong understanding of these relationships. Organizations are encouraged to delve deeper into these metrics to identify additional patterns and refine their strategies for fostering a supportive workplace culture.

#### Association rule sorted by confidence

Figures 6 and  7 the top association rules ranked by confidence, capturing the conditional likelihood of a consequent given its antecedent. Unlike support, which reflects how frequently a rule appears in the dataset, confidence evaluates the reliability of the association and indicates how consistently an outcome follows a given condition.

The rule “phys_health_interview: No $\rightarrow$ mental_health_interview: No” exhibits the highest confidence value, indicating that employees who refrain from discussing physical health during interviews are highly likely to also avoid discussing mental health. This pattern suggests a broader reluctance to disclose health-related concerns in formal recruitment settings, potentially driven by perceived stigma or fear of negative evaluation.

From an organizational perspective, high-confidence rules are particularly valuable because they identify reliable behavioral patterns that can inform targeted interventions. For example, structured interview guidelines, interviewer training, and explicit communication about confidentiality may help reduce barriers to health-related disclosure. Evaluating confidence alongside support enables organizations to prioritize interventions that are not only common but also consistently predictive of employee behavior, thereby supporting more effective and evidence-based mental health policies^29,61^.

#### Association rules sorted by lift

Figures 8 and  9 presents the top association rules ranked by lift, which measures the strength of an association relative to statistical independence. A lift value greater than one indicates that the antecedent and consequent co-occur more frequently than would be expected by chance, highlighting non-random and potentially meaningful relationships.

The rule “seek_help: Yes $\rightarrow$ wellness_program: Yes” has the highest lift value of 3.54. This suggests that employees who seek help are more likely to be aware of wellness programs provided by their employers. Similarly, the rule “seek_help: Yes $\rightarrow$ benefits: Yes” shows a lift of 2.16, suggesting that employees who seek mental health support are more likely to have access to mental health benefits. These findings are significant as they highlight the role of workplace resources in supporting employee mental health. Other high-lift rules, such as “supervisor: No $\rightarrow$ coworkers: No” and “no_employees: 1-5 $\rightarrow$ benefits: No”, reveal structural and organizational factors associated with limited mental health support. In particular, these patterns suggest that weak supervisory and peer support, as well as smaller organizational size, may be linked to reduced availability of mental health benefits.

From an organizational perspective, lift shows whether any condition actually makes the outcome more likely compared to its normal occurrence in the organization. As a result, lift helps identify intervention points that represent meaningful leverage for action. Such insights can guide targeted initiatives, for example by strengthening managerial support structures or expanding wellness resources in smaller organizations, where impactful but less common relationships might otherwise be overlooked.

**Fig. 10:**
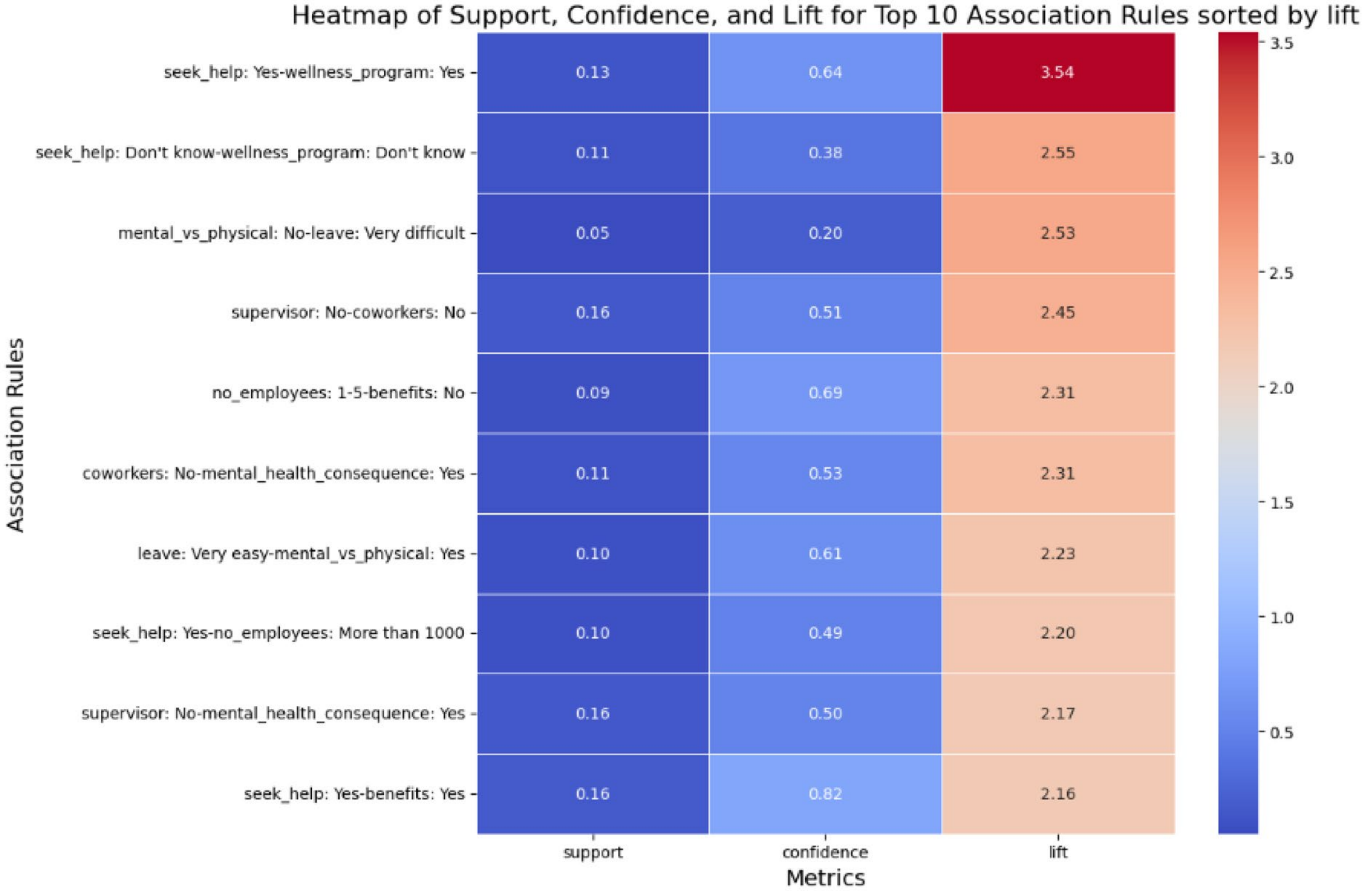
Heatmap of support, confidence, and lift values for the top 10 association rules ranked by lift. Each row corresponds to an association rule, while columns represent support, confidence, and lift metrics. Color intensity reflects the magnitude of each metric, enabling comparative interpretation of rule prevalence, conditional strength, and association strength.

The heatmap^62^ presented in Fig. 10 visualizes the top 10 association rules sorted by lift. The support values for most rules are relatively low, indicating that these associations occur infrequently across the dataset. However, the confidence values are moderate to high, suggesting a strong likelihood that the antecedent leads to the consequent in most cases. The lift values, which are the primary focus of this heatmap, range from 2.16 to 3.54. These values highlight a positive relationship, where the occurrence of the antecedent significantly increases the likelihood of the consequent, demonstrating the strength of these associations.

### Network graph analysis

**Fig. 11:**
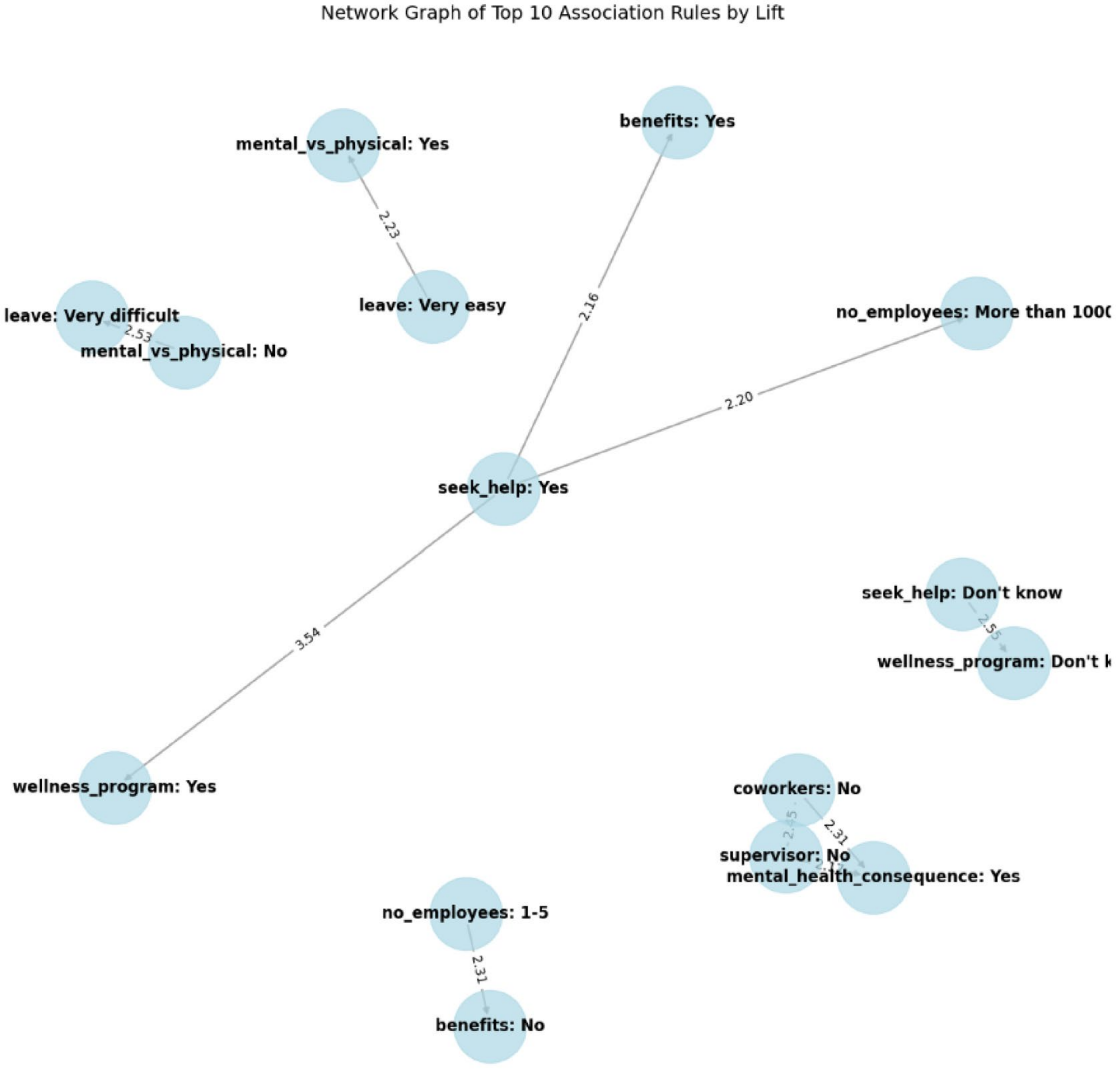
Each node in the graph represents a survey variable (or combination of responses), and the edges between them signify the lift values and associations uncovered by the frequent responses mining and association rule generation process.

The network graph^63,64^ in Fig. 11 visually represents the top association rules based on the Lift metric, showcasing the relationships between different survey responses in terms of their co-occurrence and dependency. The central group of four nodes (seek_help: Yes, benefits: Yes, no_employees: More than 1000, and wellness_program: Yes) highlights a key pattern: employees who seek help for mental health issues are more likely to work in large organizations that offer mental health benefits and wellness programs. This indicates a strong infrastructure of mental health support in large organizations that encourages employees to take advantage of available resources. Similarly, the association between no_employees: 1-5 and benefits: No, with a Lift value of 2.31, indicates a higher likelihood that employees in smaller companies are not offered mental health support, which can impact their well-being and productivity. Additionally, the relationships among these nodes emphasize the importance of both the organizational size and the wellness programs offered in promoting mental health awareness and support, which ultimately encourages employees to seek help when needed. This is critical for organizations aiming to improve employee well-being and create a mentally healthy workplace culture.

Also, the association between seek_help: Don’t know and wellness_program: Don’t know, with a Lift value of 2.50, reveals that employees who are unsure about mental health support are also likely to be uninformed about the resources provided by their organization. Overall, this network graph analysis is effective in providing a visual representation of the complex interrelationships between various workplace factors affecting mental health. By identifying strong connections between these factors, the analysis offers valuable insights for organizations to improve their policies and practices related to employee well-being. The analysis helps in recognizing gaps in employee support systems, such as the need for better communication about wellness programs and benefit. These insights are crucial for creating a more supportive and mentally healthy work environment.

#### Association rule metrics analysis: key findings in workplace mental health

The metrics of support, confidence, and lift provide complementary insights into the relationships between various mental health factors in the workplace. Firstly, support helps identify which combinations of employee responses are frequent across the dataset. For instance, a high support value indicates that these factors co-occur frequently in the dataset, suggesting that they are commonly experienced by employees. However, while support helps us understand the prevalence of certain combinations, it does not reveal how strongly these factors influence each other, which is why it is paired with other metrics.

Secondly, confidence, in this context, measures the likelihood. For example, if an employee reports mental health issues interfering with their work, confidence tells us how likely it is that they will also seek treatment. This metric allows us to assess the strength of the relationship between the antecedent and the consequent. However, it can be misleading because it does not account for the overall frequency of the consequent in the dataset, meaning it might overemphasize relationships that are less common overall.

Lastly, lift is particularly valuable in our research as it helps account for the frequency of both the antecedent and consequent independently, thereby providing a more accurate picture of their association. A lift greater than 1, for instance, indicates that the combination of factors occurs more frequently than would be expected by chance, implying that these factors are positively related. In the context of mental health at the workplace, this helps identify which combinations of responses truly influence each other beyond mere co-occurrence. By using all three metrics together, our research gains a comprehensive understanding of how different mental health-related factors are interconnected, revealing not just frequent patterns but also the strength and significance of their relationships, which can inform better workplace wellness policies.

### Technical evaluation setup and baseline comparison

To provide a controlled and technically consistent comparison, the proposed ECLAT-based framework was evaluated against two established association rule mining algorithms, namely Apriori and FP-Growth. All methods were executed on the same transaction dataset using identical minimum support and minimum confidence thresholds, fixed at 0.05 and 0.60, respectively. To ensure both computational tractability and methodological fairness, the maximum itemset length was restricted to two (i.e., pairwise association rules) for all algorithms.

This configuration serves two technical objectives. First, pairwise association rules are widely regarded as the most interpretable and actionable form of co-occurrence patterns in survey-based organizational analytics, where higher-order interactions often lack clear managerial meaning. Second, constraining the rule length mitigates the combinatorial explosion associated with candidate generation in breadth-first search–based methods such as Apriori, enabling meaningful and stable comparisons of runtime and memory consumption under matched conditions.

All evaluated algorithms recover the same association rules under identical support, confidence, and rule-length constraints. Consequently, rule-quality metrics such as support, confidence, and lift are identical across methods. Consequently, differences in performance arise solely from algorithmic design choices rather than variations in mining outcomes. The comparative analysis therefore focuses on computational efficiency, using runtime and memory usage as primary evaluation criteria.

**Table 4:** Comprehensive Baseline Comparison.

**Algorithm**	**MinSup**	**MinConf**	**MaxLen**	**Runtime (s)**	**Memory (MB)**	**Rule Count**	**Avg Support**	**Avg Confidence**	**Avg Lift**
Apriori	0.05	0.60	2	0.27	6.00	475	0.2549	0.7373	1.1058
FP-Growth	0.05	0.60	2	53.33	31.69	475	0.2549	0.7373	1.1058
ECLAT (Proposed)	0.05	0.60	2	**0.0005**	**0.018**	475	0.2549	0.7373	1.1058

Table 4 reports the computational efficiency of the evaluated algorithms. While Apriori and FP-Growth require substantially greater computational resources, the proposed ECLAT framework achieves orders-of-magnitude reductions in runtime and memory usage under identical rule-generation constraints. These efficiency gains are attributable to ECLAT’s vertical data representation and intersection-based mining strategy, which eliminates repeated database scans and candidate enumeration. In contrast, Apriori relies on iterative candidate generation, and FP-Growth incurs additional overhead from recursive tree construction and traversal. The results demonstrate that the proposed ECLAT-based framework delivers superior computational scalability while preserving equivalent analytical outcomes.

### Real world application

To implement ECLAT effectively, organizations can integrate the algorithm into their data analysis pipeline by first collecting comprehensive survey data from employees, ensuring that the dataset includes relevant factors such as participation in wellness programs, treatment-seeking behaviors, workplace stigma, and other mental health-related variables. Importantly, the analytical procedure described in this study is not tied to a particular dataset or time period; rather, it is readily transferable to survey data collected across different organizational settings, industries, and temporal contexts.

Once the data is collected, ECLAT can be applied to identify frequent responses and association rules, revealing authentic and reliable patterns in employee mental health responses. These insights can then be used to shape policies, optimize wellness initiatives, and address workplace challenges, leading to a more supportive environment for mental well-being. By embedding this algorithm into the ongoing data analysis process, organizations can continuously monitor and refine their strategies to improve employee mental health, ultimately fostering a healthier, more productive workforce. Organizations can proactively identify areas for improvement and take evidence-based actions to enhance employee well-being and overall organizational success.

### Limitations and future research

While the proposed ECLAT-based framework is designed to be transferable across organizational contexts, a limitation of the present study lies in the temporal scope of the empirical dataset used for demonstration. The survey data reflects workplace mental health conditions at a specific point in time and does not capture subsequent structural changes in work practices. Future research can apply the same analytical framework to post-pandemic or longitudinal datasets to examine how association patterns evolve across different phases of workplace transformation. Such extensions would further enhance the generalizability and empirical scope of the findings while preserving the methodological contributions of this study.

## Conclusion

The research demonstrates the novel and effective application of the ECLAT algorithm in analyzing employee mental health data within the workplace. By uncovering frequent survey questions responses and generating meaningful association rules, the study provides valuable insights into how various mental health-related factors, such as wellness programs, treatment-seeking behaviors, and workplace stigma, influence employee well-being. The results suggest that certain factors, such as the availability of mental health benefits and the size of the organization, have a significant impact on employees’ mental health perceptions and behaviors. Moreover, the use of support, confidence, and lift metrics in association rule mining has enabled a deeper understanding of the relationships between these factors. This research also emphasizes the importance of workplace policies and practices in shaping employees’ mental health experiences. The insights gained from this analysis can serve as a foundation for organizations to improve mental health support, tailor wellness programs to the specific needs of employees, and address stigma related to mental health discussions. Ultimately, by promoting a more supportive and mentally healthy work environment, organizations can enhance employee engagement, productivity, and overall organizational success. The findings highlight the potential for data-driven approaches, such as frequent responses mining and association rule discovery, to inform workplace well-being initiatives and contribute to the development of healthier, more supportive work environments.

## Data Availability

The data supporting the findings of this study are available from the corresponding author upon reasonable request.
